# Supplementary material for: Pharmacologic AMPK Activation Extends Lifespan in C. elegans and Improves Aspects of Healthspan in Mice
Source: bioRxiv. 2026 Apr 29:2026.04.21.719899. Preprint. [Version 2] doi: 10.64898/2026.04.21.719899 (PMC13131576; doi:10.64898/2026.04.21.719899)
Supplement: Supplement 2 [file NIHPP2026.04.21.719899v2-supplement-2.pdf]

bioRxiv preprint doi: https://doi.org/10.64898/2026.04.21.719899; this version posted April 29, 2026. The copyright holder for this preprint (which was not certified by peer review) is the author/funder, who has granted bioRxiv a license to display the preprint in perpetuity. It is made available under aCC-BY 4.0 International license.

Supplemental Figures

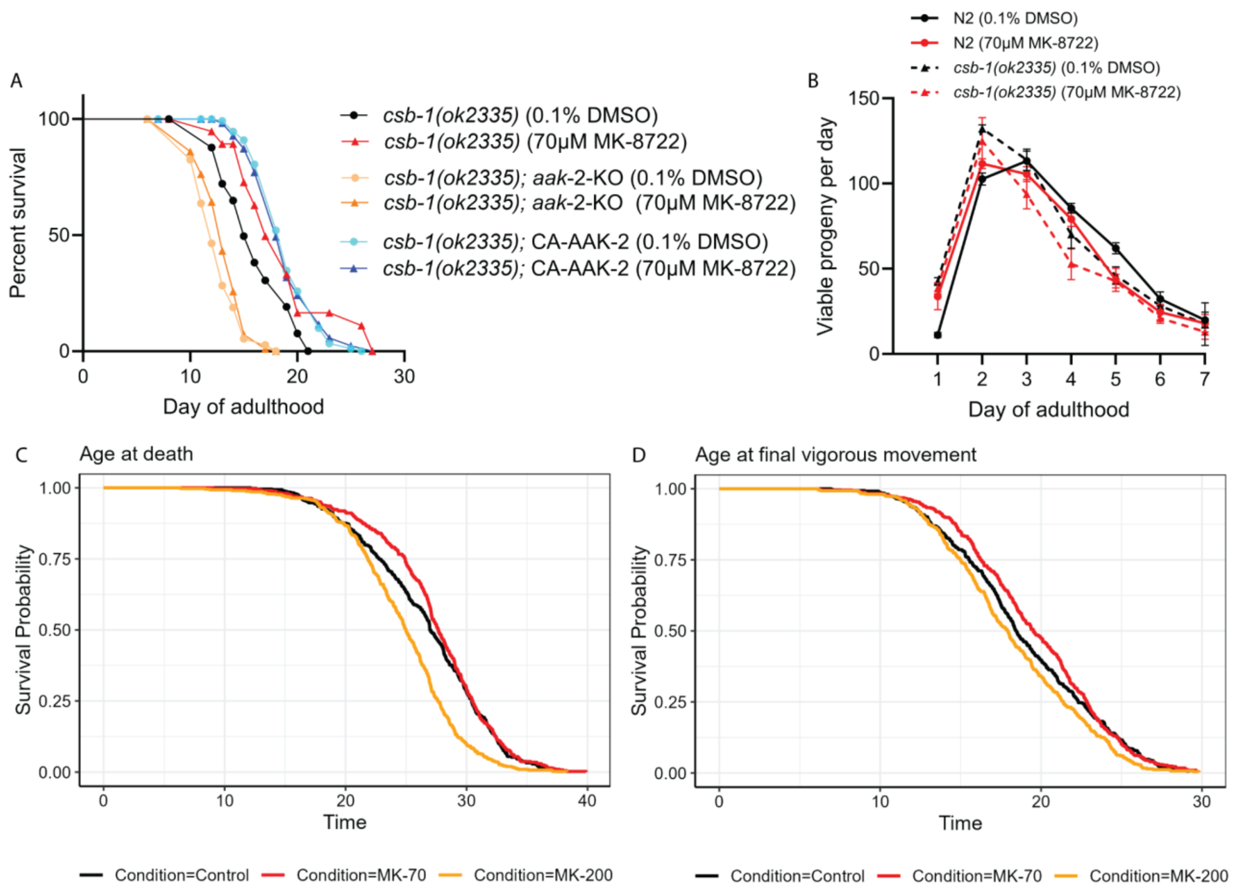

Supplementary Figure 1: **A)** Total viable progeny per day from day 1 until day 7 of adulthood of wild-type and *csb-1(ok2335)* hermaphrodites mated to males of the same genotype and treated with vehicle or 70 μM MK-8722. **B)** Lifespans of *csb-1(ok2335)* mutants, *csb-1(ok2335)* mutants crossed with *aak-2* null mutants and *csb-1(ok2335)* mutants expressing constitutively active AAK-2 and treated with vehicle or 70 μM MK-8722. **C)** Survival curves for wild-type N2 *C. elegans* treated with vehicle control or MK-8722 at 70 μM or 200 μM with analysis and quantification performed using an automated image-based Worm Lifespan Machine protocol. **D)** Quantitation of age of final vigorous movement from the experiment described in **C.** For manual lifespans at least 60 worms per condition were used. Detailed statistics and sample sizes are in Supplemental Workbook 1 and methods.

bioRxiv preprint doi: https://doi.org/10.64898/2026.04.21.719899; this version posted April 29, 2026. The copyright holder for this preprint (which was not certified by peer review) is the author/funder, who has granted bioRxiv a license to display the preprint in perpetuity. It is made available under aCC-BY 4.0 International license.

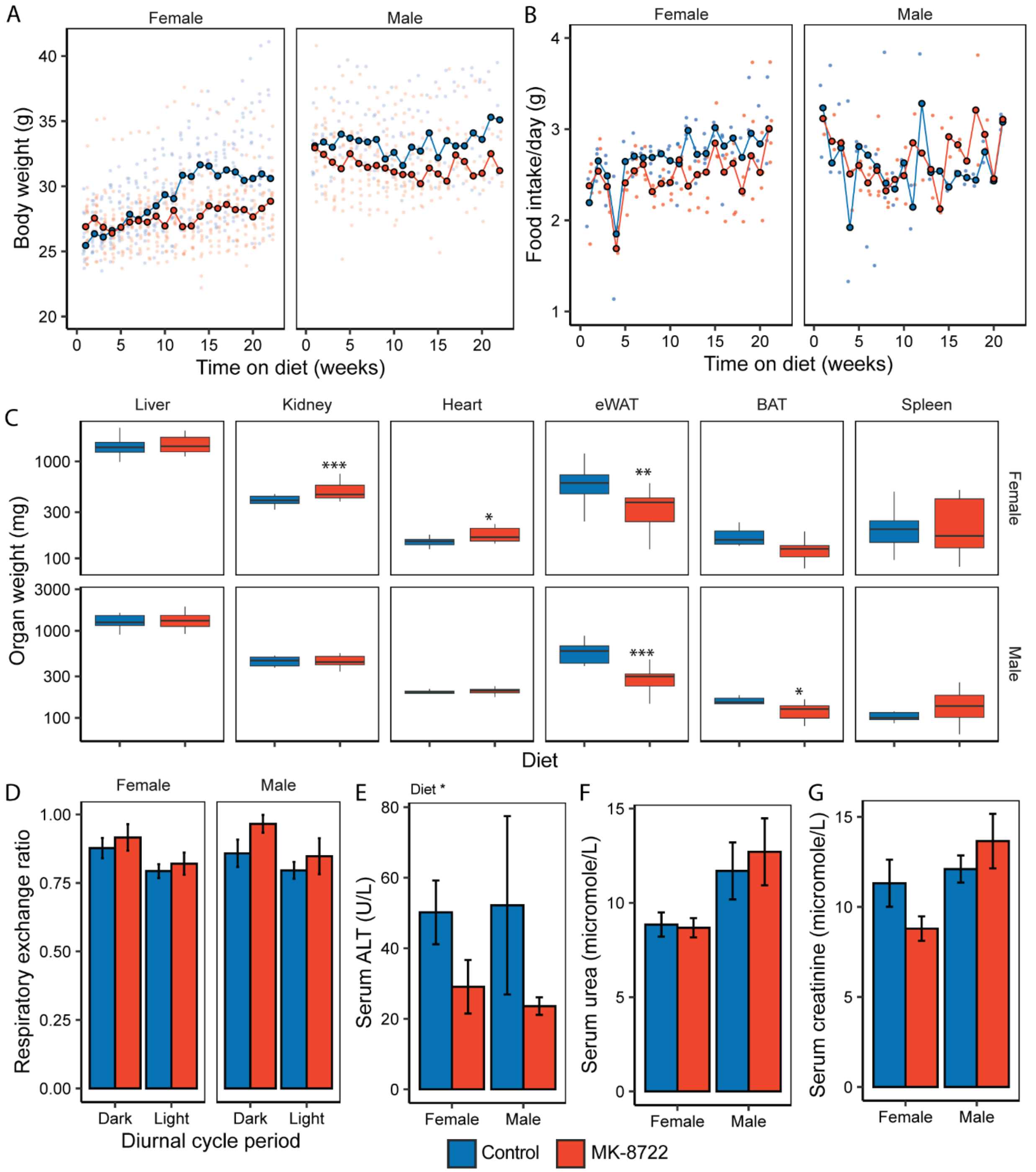

Supplementary Figure 2: A) Body weight trajectories of control and MK-treated female and male mice over the six-month study period. **B)** Food intake of control and MK-treated female (left) and male (right) mice over the six-month study period. **C)** Organ weight of control and MK-treated female and male mice following sacrifice at the end of the six-month study period. **D)** Respiratory exchange ratio of control and MK-treated female and male mice after approximately 5 months of treatment. Values are the per-mouse median across three days of measurement for the 12-hour dark and 12-hour light cycles. **E)** Serum alanine aminotransferase (ALT), **F)** urea, and **G)**

bioRxiv preprint doi: https://doi.org/10.64898/2026.04.21.719899; this version posted April 29, 2026. The copyright holder for this preprint (which was not certified by peer review) is the author/funder, who has granted bioRxiv a license to display the preprint in perpetuity. It is made available under aCC-BY 4.0 International license.

creatinine concentrations from serum collected at the time of sacrifice. For **C)** asterisks indicate significance from non-parametric t-test comparing MK to Control within sex. For E-G asterisks indicate term significance from ANOVA including sex and diet. *, **, *** indicate $P<0.05$, 0.01, 0.001 respectively. Group N was 7-20, as detailed in methods.

bioRxiv preprint doi: https://doi.org/10.64898/2026.04.21.719899; this version posted April 29, 2026. The copyright holder for this preprint (which was not certified by peer review) is the author/funder, who has granted bioRxiv a license to display the preprint in perpetuity. It is made available under aCC-BY 4.0 International license.

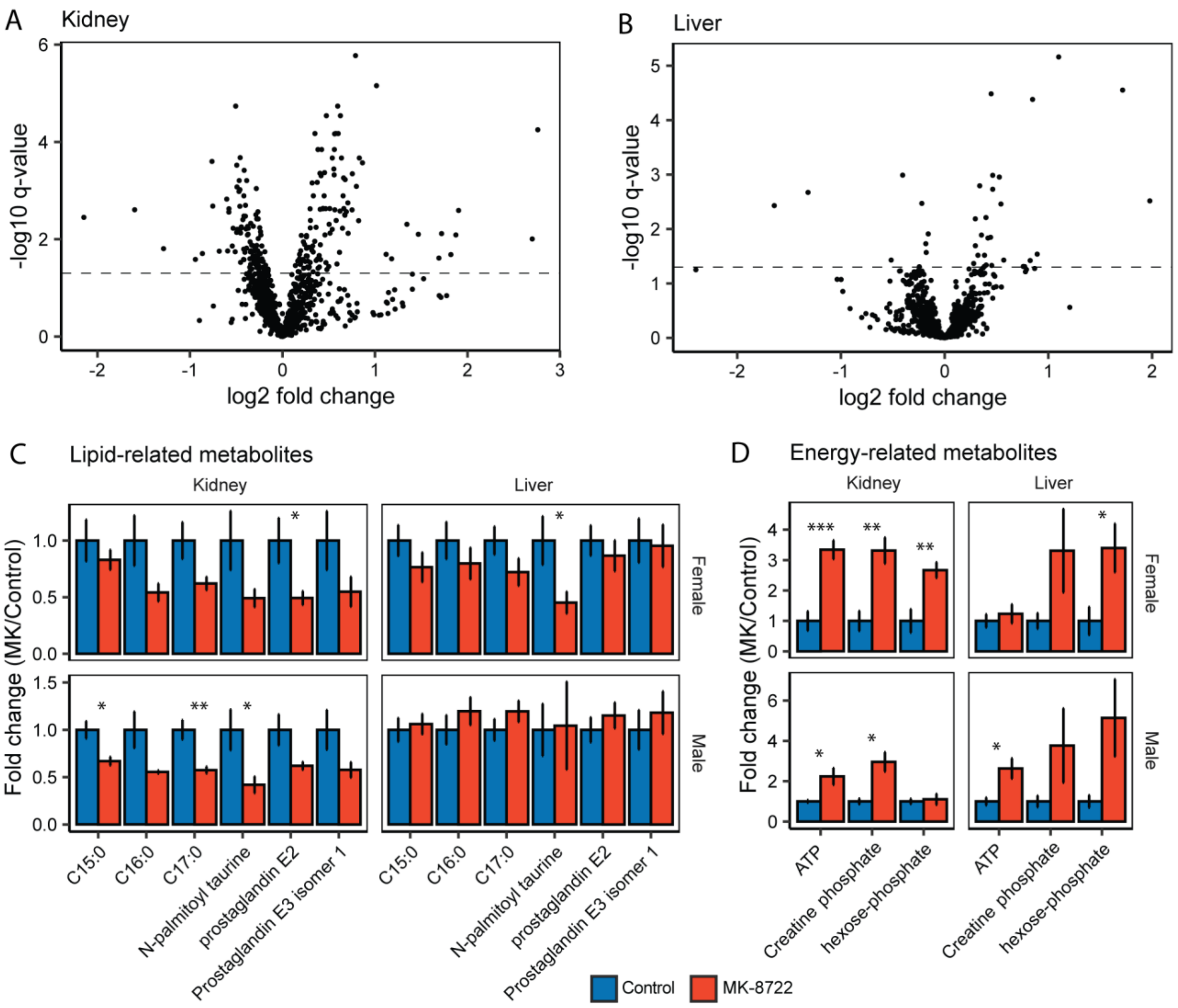

Supplementary Figure 3. A) Volcano plot showing log2 fold change (MK/Control) and -log10 FDR-corrected p-values for polar metabolites measured in the kidney and **B)** liver of male and female mice treated with MK or Control for 6 months from 18-24 months of age. **C)** Fold changes vs Control treated mice of lipid-related and **D)** energy production-related metabolites that showed a significant diet-effect (MK vs Control) in statistical models assessing effects of diet, sex and MK-by-sex interaction in kidney and liver metabolomics data. Asterisks *, **, *** indicate P<0.05, 0.01, 0.001 respectively from a non-parametric t-test comparing MK to Control within sex.

bioRxiv preprint doi: https://doi.org/10.64898/2026.04.21.719899; this version posted April 29, 2026. The copyright holder for this preprint (which was not certified by peer review) is the author/funder, who has granted bioRxiv a license to display the preprint in perpetuity. It is made available under aCC-BY 4.0 International license.

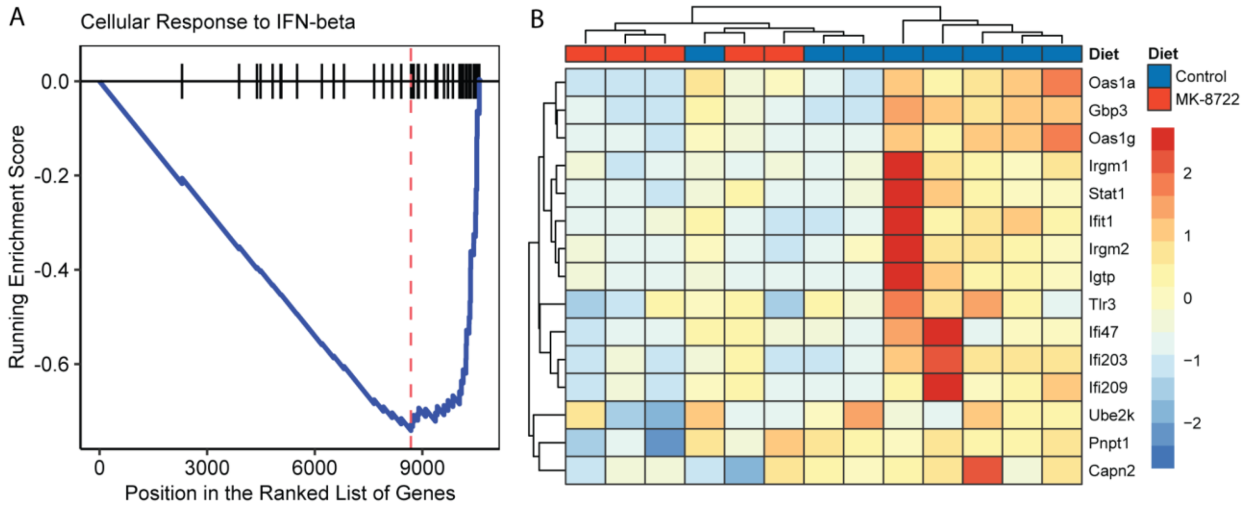

Supplementary Figure 4. A) GSEA plot for the GO pathway "Cellular Response to Interferon-Beta". **B)** Heatmap of all nominally significantly expressed genes in the GO pathway "Cellular Response to Interferon-Beta".

bioRxiv preprint doi: https://doi.org/10.64898/2026.04.21.719899; this version posted April 29, 2026. The copyright holder for this preprint (which was not certified by peer review) is the author/funder, who has granted bioRxiv a license to display the preprint in perpetuity. It is made available under aCC-BY 4.0 International license.

Table S1. Electrocardiogram parameters in female mice

	mo00			mo03			mo06		
**Characteristic**	**CON** N = 14[1]	**MK** N = 20[1]	**p-value**[2]	**CON** N = 14[1]	**MK** N = 20[1]	**p-value**[2]	**CON** N = 14[1]	**MK** N = 20[1]	**p-value**[3]
HR	766 (738, 783)	755 (735, 762)	0.083	751 (735, 758)	735 (725, 746)	0.034	746 (737, 764)	739 (722, 749)	0.2
HRV	40 (29, 58)	30 (14, 70)	0.6	40 (21, 47)	42 (20, 49)	>0.9	62 (32, 96)	83 (46, 110)	0.4
CV	5.3 (3.7, 7.7)	3.9 (1.9, 9.2)	0.7	5.11 (2.97, 6.24)	5.62 (2.76, 6.63)	>0.9	8.2 (4.2, 12.9)	11.2 (6.2, 14.3)	0.3
RR	78.83 (77.28, 81.62)	80.09 (79.32, 82.17)	0.074	80.61 (79.27, 81.83)	82.17 (80.80, 83.48)	0.022	81.53 (80.15, 81.72)	83.00 (81.33, 84.91)	0.069
PQ	15.70 (14.59, 16.96)	17.73 (16.50, 18.51)	0.015	15.78 (14.83, 17.80)	17.40 (16.00, 18.82)	0.12	15.39 (14.07, 17.59)	17.72 (16.80, 18.78)	0.023
PR	21.80 (20.93, 23.35)	24.03 (22.65, 25.17)	0.009	22.32 (21.23, 24.04)	24.00 (22.32, 24.85)	0.092	21.68 (21.15, 24.16)	24.11 (23.00, 25.13)	0.016
QRS	11.08 (10.48, 11.37)	10.80 (10.63, 11.49)	>0.9	11.10 (10.70, 11.33)	11.14 (10.42, 11.40)	0.8	11.02 (10.67, 11.70)	10.73 (10.60, 11.10)	0.13
QT	39.28 (38.82, 41.03)	40.33 (39.29, 41.18)	0.2	40.29 (39.91, 40.82)	40.70 (40.16, 42.15)	0.13	39.98 (39.32, 40.92)	40.81 (40.47, 41.40)	0.2
ST	28.98 (28.30, 30.40)	29.73 (28.94, 30.89)	0.2	29.73 (29.42, 30.85)	30.57 (29.77, 31.60)	0.13	29.75 (28.07, 30.46)	30.72 (30.22, 31.05)	0.032
QTC	44.67 (44.07, 46.10)	45.29 (44.11, 45.86)	0.7	45.18 (44.55, 45.57)	45.50 (44.76, 46.47)	0.4	44.73 (44.04, 45.32)	45.23 (44.23, 45.55)	0.5
QTdisp	25.5 (21.9, 34.4)	26.7 (22.2, 31.2)	0.6	26.3 (23.1, 30.4)	27.1 (24.2, 28.5)	0.7	34 (25, 42)	33 (27, 42)	>0.9
QTCdisp	30 (26, 38)	30 (25, 38)	0.5	33 (28, 37)	33 (28, 36)	0.9	40 (30, 47)	44 (31, 50)	0.7
SRamp	0.33 (0.25, 0.38)	0.28 (0.21, 0.32)	0.11	0.31 (0.23, 0.33)	0.31 (0.28, 0.38)	0.4	0.30 (0.24, 0.33)	0.33 (0.30, 0.36)	0.12
Ramp	0.23 (0.20, 0.31)	0.21 (0.18, 0.28)	0.2	0.24 (0.20, 0.26)	0.23 (0.23, 0.29)	0.3	0.24 (0.18, 0.29)	0.27 (0.22, 0.29)	0.3
Power	10 (3, 28)	5 (1, 24)	0.4	12 (7, 23)	7 (1, 14)	0.3	27 (5, 56)	45 (5, 96)	0.6
lowPower	3 (0, 8)	2 (0, 5)	0.5	2.6 (0.8, 6.0)	0.9 (0.6, 6.5)	0.5	8 (2, 24)	20 (1, 40)	0.7
highPower	1.8 (1.2, 8.5)	0.8 (0.3, 6.5)	0.2	4.2 (2.0, 8.7)	2.5 (0.1, 4.1)	0.11	9 (1, 15)	10 (1, 22)	0.8
lowHighRatio	2.16 (1.59, 3.18)	4.03 (2.48, 5.99)	0.023	3 (2, 5)	7 (5, 10)	0.003	1.7 (1.0, 3.1)	2.8 (1.9, 5.8)	0.3
LF	0.39 (0.37, 0.45)	0.46 (0.41, 0.54)	0.12	0.41 (0.33, 0.50)	0.49 (0.44, 0.64)	0.010	0.37 (0.27, 0.44)	0.41 (0.35, 0.47)	0.3
HF	0.28 (0.22, 0.30)	0.22 (0.17, 0.28)	0.13	0.31 (0.22, 0.38)	0.21 (0.13, 0.26)	0.009	0.29 (0.20, 0.37)	0.27 (0.21, 0.31)	0.6
rMSSD	5.2 (4.6, 11.2)	3.9 (2.0, 9.6)	0.2	5.10 (3.51, 6.50)	3.58 (2.37, 7.08)	0.3	8.1 (3.9, 12.7)	11.1 (6.0, 13.9)	0.7
pNN50	4.5 (2.6, 7.8)	2.1 (1.0, 7.0)	0.3	3.0 (1.9, 6.1)	3.0 (1.1, 5.3)	0.7	5 (3, 9)	8 (4, 15)	0.3

[1] Median (Q1, Q3)

[2] Wilcoxon rank sum test; Wilcoxon rank sum exact test

[3] Wilcoxon rank sum exact test; Wilcoxon rank sum test

bioRxiv preprint doi: https://doi.org/10.64898/2026.04.21.719899; this version posted April 29, 2026. The copyright holder for this preprint (which was not certified by peer review) is the author/funder, who has granted bioRxiv a license to display the preprint in perpetuity. It is made available under aCC-BY 4.0 International license.

Table S2. Electrocardiogram parameters in male mice

	mo00			mo03			mo06		
**Characteristic**	**CON** N = 7[1]	**MK** N = 14[1]	**p-value**[2]	**CON** N = 7[1]	**MK** N = 14[1]	**p-value**[2]	**CON** N = 7[1]	**MK** N = 14[1]	**p-value**[3]
HR	755 (745, 784)	757 (744, 774)	>0.9	751 (738, 772)	741 (718, 750)	0.14	758 (747, 774)	744 (740, 751)	0.073
HRV	49 (30, 83)	77 (52, 85)	0.3	36 (19, 64)	44 (21, 87)	0.6	99 (68, 100)	87 (42, 136)	0.7
CV	6.6 (4.1, 11.0)	10.7 (6.8, 11.3)	0.3	4.8 (2.6, 8.3)	6.0 (2.9, 11.9)	0.5	13 (9, 13)	11 (6, 18)	0.7
RR	80.07 (77.60, 80.82)	79.77 (78.50, 81.83)	0.6	80.40 (78.33, 81.64)	81.56 (80.60, 84.56)	0.10	81.17 (78.62, 82.34)	81.78 (80.48, 84.20)	0.3
PQ	17.10 (15.13, 19.80)	16.69 (14.99, 18.03)	0.5	16.59 (15.73, 17.78)	17.20 (16.26, 18.70)	0.4	16.02 (14.24, 18.20)	17.22 (15.58, 17.95)	0.9
PR	22.97 (21.80, 25.70)	22.54 (21.63, 23.78)	0.4	22.29 (22.08, 24.28)	23.23 (22.07, 24.36)	0.6	22.65 (20.82, 23.62)	23.13 (21.80, 24.23)	0.6
QRS	10.45 (10.13, 11.14)	10.75 (10.25, 11.14)	0.6	10.88 (10.07, 11.05)	10.13 (9.80, 10.62)	0.2	10.79 (10.48, 10.97)	11.06 (10.67, 11.26)	0.4
QT	39.56 (39.30, 39.98)	40.31 (39.27, 41.40)	0.5	40.30 (39.00, 40.85)	39.68 (39.48, 41.45)	>0.9	39.78 (39.68, 40.85)	40.55 (39.90, 41.75)	0.4
ST	29.80 (28.94, 30.03)	29.66 (28.81, 31.20)	0.8	29.55 (29.44, 30.65)	30.38 (29.95, 31.33)	0.3	29.73 (29.33, 30.24)	30.15 (29.43, 30.40)	0.4
QTC	44.53 (44.10, 45.26)	45.08 (44.34, 45.25)	0.6	45.04 (43.88, 45.30)	44.28 (43.56, 45.41)	0.8	44.80 (44.30, 45.38)	44.94 (44.75, 46.47)	0.7
QTdisp	28 (23, 31)	36 (33, 45)	0.081	29 (26, 37)	32 (29, 35)	0.7	45 (41, 50)	45 (27, 56)	0.9
QTCdisp	35 (30, 42)	43 (39, 52)	0.11	34 (31, 44)	36 (32, 46)	0.8	54 (48, 63)	52 (33, 64)	0.7
SRamp	0.31 (0.23, 0.35)	0.29 (0.19, 0.32)	0.5	0.26 (0.19, 0.29)	0.29 (0.18, 0.36)	0.5	0.26 (0.25, 0.27)	0.19 (0.17, 0.22)	0.3
Ramp	0.21 (0.17, 0.31)	0.20 (0.16, 0.27)	0.3	0.22 (0.18, 0.24)	0.20 (0.17, 0.25)	>0.9	0.22 (0.20, 0.23)	0.18 (0.15, 0.23)	0.3
Power	9 (5, 13)	26 (22, 49)	0.011	3 (1, 27)	11 (3, 24)	0.8	72 (58, 82)	94 (9, 157)	0.8
lowPower	2 (1, 3)	10 (3, 13)	0.046	2.0 (0.2, 18.3)	3.7 (0.8, 8.3)	>0.9	24 (17, 31)	38 (3, 71)	0.6
highPower	2 (1, 4)	11 (5, 16)	0.037	1.3 (0.3, 5.2)	3.6 (1.1, 8.3)	0.4	15 (11, 31)	22 (3, 35)	0.9
lowHighRatio	1.89 (0.45, 2.55)	1.67 (1.17, 2.51)	0.7	2.64 (2.10, 4.70)	1.63 (0.84, 3.33)	0.12	1.50 (0.81, 2.22)	1.98 (1.67, 2.67)	0.15
LF	0.37 (0.17, 0.42)	0.32 (0.26, 0.38)	>0.9	0.41 (0.33, 0.48)	0.34 (0.23, 0.44)	0.3	0.30 (0.23, 0.40)	0.36 (0.33, 0.46)	0.15
HF	0.35 (0.28, 0.40)	0.31 (0.28, 0.38)	0.8	0.30 (0.22, 0.36)	0.31 (0.22, 0.39)	0.7	0.33 (0.27, 0.38)	0.29 (0.27, 0.32)	0.3
rMSSD	6.6 (5.2, 10.6)	10.6 (7.6, 12.6)	0.11	5.5 (2.7, 8.5)	6.4 (3.3, 11.4)	0.7	14 (12, 18)	17 (4, 24)	>0.9
pNN50	5 (2, 7)	7 (5, 10)	0.3	3.0 (1.0, 6.4)	2.8 (1.1, 6.9)	>0.9	17 (6, 26)	14 (4, 18)	0.6

[1] Median (Q1, Q3)

[2] Wilcoxon rank sum test; Wilcoxon rank sum exact test

[3] Wilcoxon rank sum exact test; Wilcoxon rank sum test
